# Creating the necessary infrastructure for a trauma-informed system of care for children and youth

**DOI:** 10.3389/fpsyg.2023.1129197

**Published:** 2023-07-11

**Authors:** John S. Lyons, April D. Fernando

**Affiliations:** Center for Innovation in Population Health, College of Public Health, University of Kentucky, Lexington, KY, United States

**Keywords:** trauma-informed system, Transformational Collaborative Outcomes Management, person-centered system of care, child and adolescent needs and strengths, traumatic stress symptoms, communimetrics, practice-based research, population health

## Abstract

Understanding and addressing the impact of adverse life events is an important priority in the design of helping systems. However, creating trauma-informed systems requires efforts to embed effective trauma-informed work in routine practice. This article discusses a model for developing trauma-informed systems using the Transformational Collaborative Outcomes Management (TCOM) framework, a strategy for engineering person-centered care. Person-centered care is naturally congruent with trauma-informed care. We describe the initial stages of implementation of a trauma-informed standardized assessment process to support the sustained evolution of trauma-informed care. Distinguishing between traumatic experiences and traumatic stress is fundamental to an effective trauma-informed system. We describe two sets of analyses—one in a statewide child welfare system and the other in a statewide behavioral health system. These projects found opportunities in the analysis of the detection of traumatic stress based on traumatic experiences to inform practice and policy. Being trauma-informed in child welfare is distinct from being trauma-informed in behavioral health. In child welfare, it appears that a number of children are resilient in the face of traumatic experiences and do not require trauma treatment interventions. However, delayed and missed traumatic stress responses are common. In behavioral health, misses often occur among adolescents, particularly boys, who engage in acting out behavior. Opportunities for the ongoing development of trauma-informed systems using the TCOM framework are discussed.

## 1. History and context

### 1.1. A brief review of the literature

Much has been written on the importance of developing what has come to be called trauma-informed systems. Although an awareness that environmental events impacting the human condition has existed throughout recorded history, the genesis of this interest from a clinical perspective date back to research on the impact of war, initially in 1761, by the Austrian physician Josef Leopold (as cited in Trimble, 1985). This literature grew over different wars and began to include non-combat injuries. The extension of a trauma perspective to children arrived much later. In their important article “Ghosts in the Nursery,” Fraiberg et al. (1975) began to apply the principles of trauma response to children. This study was quickly followed in the 1980s by Bloom's development of the Sanctuary model (Bloom, 2017) and the establishment of diagnostic criteria for Posttraumatic Distress Disorder in DSM III by the American Psychiatric Association (1980). With the original publications on Adverse Childhood Experiences (Felitti et al., 1998) and continuing with a large body of research, it has become clear that experiencing traumatic events can lead to changes in brain function and worse outcomes in both short and long terms (Lo lacono et al., 2021). Presently, there is a broad appreciation of the impact of traumatic experiences in terms of both short- and long-term consequences (Monnat and Chandler, 2015; Petrucceli et al., 2019). There is less, but growing evidence, that these same traumatic events do not have uniform effects on all children, and recovery from the adverse impact is achievable (Campbell, 2020; Jones, 2020; Wexler, 2022).

The word “trauma” is taken from the Greek word for injury. However, outside of emergency medicine, the word has come to apply to a variety of important aspects of the overall circumstances when bad things happen. To bring clarity to the field, a consensus has evolved on distinguishing three aspects of trauma—the event, the experience, and the effect (SAMHSA, 2014; Lathan et al., 2021, Figure 1). The event refers to the original circumstances that may be traumatic. The experience refers to the individual's sensations, perception, and understanding of the event. The effects refer to the impact of these experiences on the person's health, wellbeing, and functioning. In this conceptualization, symptoms of traumatic stress are classified as an effect of the trauma experience.

**Figure 1:**
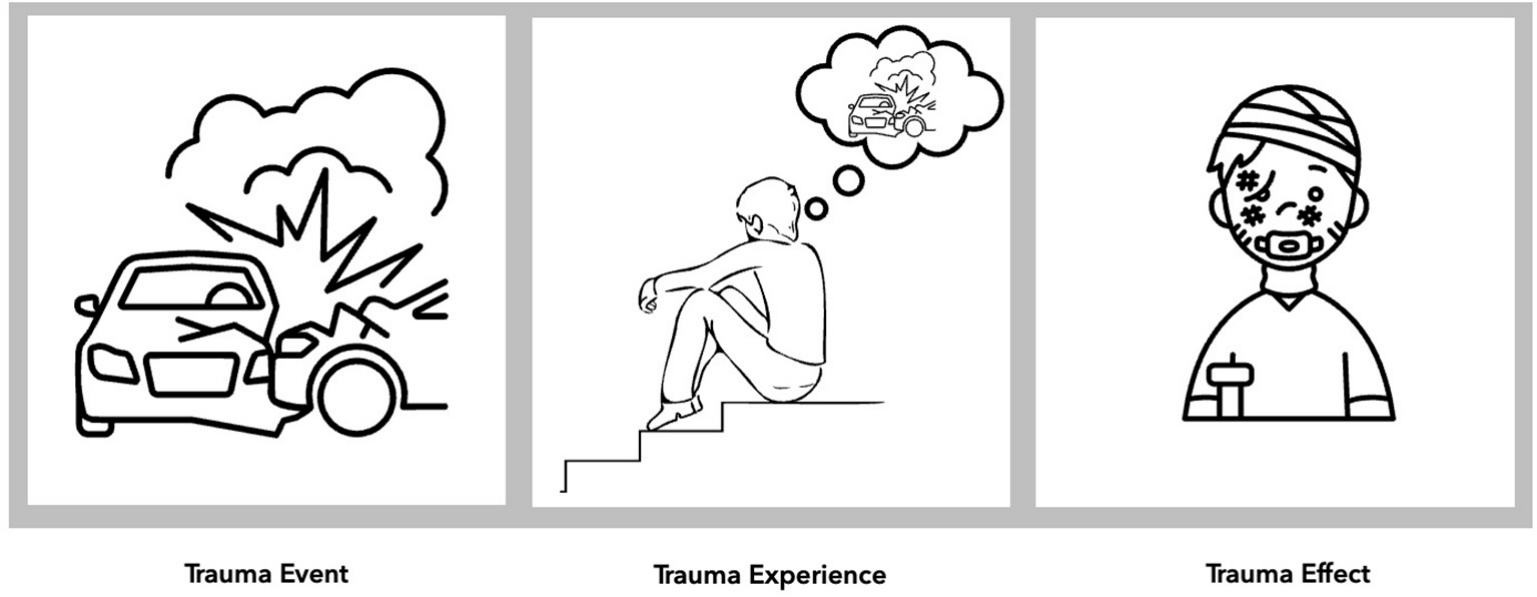
Examples of the three aspects of trauma: event, experience, and effect.

While community-based interventions might target preventing traumatic events from occurring (Tunno et al., 2021; Goodrum and Prinz, 2022), most of the attention has been on responding to the experiences and effects. Rapid response interventions in times of violence, suicide, and natural disasters are examples of interventions that attempt to target the experience to mitigate effects (e.g., Brown et al., 2017; Fraenkel and Cho, 2020; De Brier et al., 2021). Alternatively, evidence-based treatments generally target the effects (Amaya-Jackson and DeRosa, 2007; Barnett et al., 2019; Maguen et al., 2019).

There is a growing body of literature on creating trauma-informed systems (e.g., Oral et al., 2016; Clements et al., 2020); however, the majority of this literature focuses on training and educational activities. If we are to succeed in effectively creating and maintaining trauma-informed systems, more is required. We would argue that the best way to successfully build these types of systems is to structure the work itself to be trauma-informed. Simply developing a trauma-informed workforce is an important and necessary aspiration to achieve a trauma-informed system, as it is likely insufficient (Lyons, 2022). This reality is exacerbated by the rapid turnover in the workforce in many public sector systems (Beidas et al., 2016). Infrastructure that embeds trauma-informed thinking into the job roles and functions is required along with system monitoring and management approaches that support trauma-informed goals. In other words, systems must provide structures to help professionals who encourage the application of the principles of trauma-informed care (Ko et al., 2008).

## 2. Developing trauma-informed systems

Healthcare in the United States and most of the world has been organized around the treatment of diseases and disorders. This structure has created a variety of perverse incentives including (but not limited to)

° Failing up: e.g., no help is provided until a person is sick enough to meet medical necessity criteria (c.f., Singer et al., 1999; Fessel et al., 2021).
° Adverse selection: e.g., reimbursement models discourage taking on the most complex and challenging cases (c.f., Frank et al., 1997).
° Access limitations: e.g., low margins in rural areas limit the ability to create competitive healthcare marketplaces in the United States and similar jurisdictions or failure to prioritize building infrastructure in sparsely populated areas in Canada and other countries with universal health plans (c.f. Hodgkinson et al., 2017).

Alternatively, some systems do not feel comfortable identifying anything that might be perceived as negative, particularly for children. Elsewhere, we have referred to this phenomenon as the “Happy Face Syndrome” (Lyons, 2004). In the child-serving system, extreme versions of strength-based approaches sometimes verge into not providing any feedback that might be seen as negative. This strategy can be problematic to an exclusive focus on disease and dysfunction. Of course, there is a large middle ground.

Similar challenges face the development of a trauma-informed system. For example, making trauma exposure a factor to determine “eligibility” for service receipt in our present system runs the risk of making the experience of trauma function like a form of disability designation. Once present, there is no option for improvement in recovery. Individuals with trauma experiences will always be “eligible” for care regardless of whether they are experiencing symptoms of traumatic stress. While the expansion of access to care can be useful, failure to consider egress from care is a recipe for replicating the problems in our current “disease-care” approach to health and wellbeing. In Canada, for example, challenges in managing egress are a primary determinant of wait lists. To create a system that does not make traumatic experiences into a disability, we must build in exceptions for people who have traumatic experiences but do not experience traumatic stress. However, systems must recognize that when people experience multiple or substantial traumatic events, there is a good chance they will express traumatic stress and there are interventions that help.

### 2.1. Trauma-informed and person-centered

In 2001, the then Institute of Medicine (now the Academy) published the groundbreaking article “Crossing the quality chasm” which after inventorying all the challenges facing the US healthcare system recommended person-centered care as one solution. The idea of making people full partners in their healthcare is very congruent with the person-centered design movement in the product development sector and made popular by Apple and others (c.f., Eckler, 2016). Since healthcare systems have historically been designed with and for the convenience and benefit of healthcare providers, a shift to include the people who use healthcare in this design process is welcome and, sadly, overdue. We believe a person-centered approach opens the greatest possibilities for creating and sustaining effective trauma-informed systems.

The specific conceptual framework that we propose as relevant to engineering a trauma-informed system is Transformational Collaborative Outcomes Management (TCOM, Lyons, 2004, 2022). The TCOM framework uses consensus-based assessment strategies to shift the management of systems away from compliance and service delivery to a focus on engagement, collaborative helping, and personal change. The core values and guiding and operating principles of TCOM underlie a person-centered approach.

#### 2.1.1. Core values

1. Human serving systems and enterprises have a primary mandate of facilitating and supporting personal change (i.e., transformation).
2. Human serving systems and enterprises are inherently complex as a result of the number of humans involved. this diversity of aims and perspectives can only be managed through meaningful integration. integration among people is best managed through collaborative processes.
3. All partners in human-serving systems and enterprises have the responsibility for collecting, managing, and using accurate, relevant, and respectful information about the people served.

#### 2.1.2. Guiding principles

1. People have a voice and choice with regard to participating in and completing any assessments and interventions.
2. All assessments and interventions are culturally responsive and respectful.
3. All interventions should be personalized, respectful, and have demonstrable value to the people they serve.
4. Collaborative processes, respecting real-world limitations that are inclusive of individuals and families should be used for all decisions at all levels of the system.
5. Consensus on action is the primary outcome of collaborative processes.
6. Information about the people served and their personal change should always inform decision-making at all levels of the system.

#### 2.1.3. Operating principles

1. Person-centered assessments should be completed at the beginning and end of all episodes of helping and intermittently throughout extended episodes. these assessments should become the common language of the system to support a focus on the best interests of the people to be helped.
2. Everyone in the system using person-centered assessment information should be trained in the approach to ensure fluency across the system in the common language.
3. Business rules and information systems should be designed to reduce the redundancy of information to make the work and the documentation of the work as one and the same.
4. The findings of these assessments should be integrated into the operations of the helping system including planning, supervising, evaluating, and managing.

TCOM has been implemented to various degrees across North America and around the world (Lyons, 2022). In both Canada and the United States, TCOM tools have been integrated with Indigenous cultures (e.g., Doolittle and Beaucage, 2022). TCOM tools have been developed to identify the needs, strengths, and skills of children, families, and adults. Many versions exist for special populations.

## 3. The implications of a TCOM approach

One of the first steps to creating and sustaining a trauma-informed system is to identify structured assessment approaches that provide the necessary information about traumatic events and their effects. For these purposes, the assessment of trauma experiences (the individual's response to a specific traumatic event) can be quite challenging to measure. Trauma experiences are linked to factors such as interpersonal vs. non-interpersonal trauma, the dose–response relationship between the number of traumas, and the relationship between the individual and the perpetrator (Lathan et al., 2021). In addition, while an understanding of individual experiences of events might be useful in some specific trauma-informed treatment approaches, the distinction between events and experiences is less relevant for the functioning of a trauma-informed system. The most important distinction for system design is between traumatic experiences and traumatic stress symptoms. In other words, is the person experiencing traumatic stress symptoms that might be attributable to their experience of a traumatic event. Therefore, our approach separately assesses and measures traumatic experiences and symptoms of traumatic stress. In this conceptualization, the event only matters if the individual experienced it as traumatic. This focus reduces and organizes information which is important for the helping system to understand and track.

### 3.1. Person-centered assessment and measurement—Communimetrics

One of the great measurement challenges for a trauma-informed system is that talking about one's traumatic experiences can be triggering for some people and actually worsen traumatic stress (National Child Traumatic Stress Network, 2011). Traditional psychometric measurement approaches that force people to repeatedly ask the same questions in the same order with the assumption that this will reveal an otherwise unknown “truth” is simply untenable (Lyons, 2022). Psychometric measurement can be traumatizing and thus cannot be reasonably used in a trauma-informed system beyond perhaps an initial assessment/discovery process.

Instead, we have proposed a consensus-based measurement approach that organizes people's stories and carries the key common themes of those stories forward without requiring that people be repeatedly questioned about the same historical experiences. We call this approach to measurement communimetrics (Lyons, 2009, 2022). Although there are now several measures designed with this theory, the Child and Adolescent Needs and Strengths (CANS) and the Adult Needs and Strengths Assessment (ANSA) are the most widely used communimetric measures that have been adapted to support trauma-informed systems (Lyons, 2009). In this article, we will focus on the CANS since most efforts at designing and evolving trauma-informed systems have been in child-serving sectors such as child welfare, behavioral health, and schools (e.g., Akin et al., 2017).

The CANS is a widely used reliable and valid functional and clinical assessment for children and youth. Currently, the CANS is used in 33 countries around the world. In the United States, ~95% of all children and youth in public behavioral health or child welfare are touched by the CANS process. This results in the CANS being completed about 10 million times each year. The Center for Innovation in Population Health at the University of Kentucky has established a data reservoir project where millions of assessments are now linked to administrative data and other sources of information.

There are now more than 200 peer-reviewed articles from more than 50 independent research groups that use data from the CANS. Research has documented that the CANS is reliable at the item level allowing notable analytic flexibility (e.g., Anderson et al., 2003; Lyons, 2009, 2022). A substantial body of research is developing demonstrating the validity of the CANS in behavioral health, child welfare, and justice settings (Alamdari and Kelber, 2016; Juades et al., 2016; Vreeland et al., 2020; Hong et al., 2021). A part of the trauma-informed version of the CANS is a 13-item no/yes coding of trauma experiences as follows:

- No—No evidence of any trauma of this type.
- Yes—Child/youth has had experience, or there is suspicion that the child/youth has experienced this type of trauma—one incident, multiple incidents, or chronic, ongoing experiences.

In addition, the CANS has an item called “Adjustment to Trauma” which describes the identified presence of any traumatic stress symptoms. Many versions of the CANS also include a Traumatic Stress Symptoms Module containing multiple items that highlight the impact of PTSD symptomatology on a child or youth's functioning (e.g., Hyperarousal, Numbing, Avoidance, Dissociation, Re-experiencing, and Emotional and/or Physical Dysregulation). Adjustment to trauma and the specific traumatic stress symptoms are rated using the standard communimetric action levels:

0. indicates no evidence, no need for action.
1. indicates watchful waiting/prevention/further assessment.
2. indicates a need for action, the need is interfering with someone's functioning.
3. indicates a need for immediate/intensive action, the need is dangerous or disabling.

As mentioned previously, one advantage of a communimetric approach to measurement is that reliability can be found at the item level (Anderson et al., 2003). This allows the creation of versions of tools to fit different circumstances. Therefore, instead of always having to use precisely the same set of items structured in precisely the same order, which is a requirement of the valid use of psychometric measures, the CANS can be customized to fit different applications of person-centered information in complex systems.

#### 3.1.1. Training, coaching, and certification in implementing TCOM

Few would disagree that a person-centered approach is important to delivering effective care. The CANS, as the TCOM strategy that captures the story of the child, is person-centered in its approach and is aligned with principles of trauma-informed care (SAMHSA, 2014). The CANS is embedded in a care process that fully engages the children youth and families seeking help, honoring their voice, acknowledging the totality of their experiences, and engaging them in a collaborative process with respect. In concept, the CANS is fully embraced by systems, programs, and providers.

Perhaps not surprisingly, the challenge comes in embedding the CANS and TCOM into everyday practice. Good implementation starts with local ownership. We encourage the development of local trainers through a university-based Center of Excellence, Training Academy, or provider-based trainer collaboratives. We work with these local training entities to customize their CANS training curricula for their local practice models and support the trainers through learning collaborative meetings. By having local trainers, we not only adapt the approach to completing the CANS and using the information from it to elevate the local practice but also build and support TCOM champions within the system that can “speak the local practice dialect” and help others understand, appreciate, and adopt the person-centered approach.

Explicitly linking the completion of the CANS to trauma-informed care during training is critical. Trauma-informed care principles, for example, provide critical guidance on how to conduct trauma assessments, how to build collaboration and consensus, and how to conceptualize and group the actionable items to create an effective, trauma-informed plan (National Child Traumatic Stress Network, 2014). Understanding that the CANS captures the story of the young person from a trauma-informed lens also reinforces the practice of updating the information on the CANS during a re-assessment rather than completing the CANS from scratch. Simply updating the CANS avoids any risk of re-traumatization from repeated questioning of information already known and a re-assessment can become a more routine part of care, rather than a separate event. This same concept can be applied to coordinating care. When young people engage in care with various providers, sharing a completed CANS from one provider to another can facilitate the coordination of care as well as avoid the young person having to re-tell their story. Instead, the completed CANS is used to verify and communicate the individual's story while being updated with any new information.

Beyond setting the person-centered philosophy of TCOM and the use of CANS, the important aspects of initial training on the CANS is a focus on the organization of the tool, learning the vocabulary of the individual items, the action level ratings, and how to apply the action levels to items described on a case vignette. A certification test is required for all CANS users to ensure their knowledge of the individual items and how to apply the action levels. An interrater reliability correlation is calculated based on an individual's ratings on the items as compared to the preferred ratings on a test vignette which helps in establishing the individual's reliability on the CANS. Individual users must have an interrater reliability coefficient of 0.70 or higher to use the tool. Certification on the tool must be completed annually by each user to maintain reliability. These certification requirements help to maintain standardization across all users and implementations of the CANS. The worldwide average reliability of certified practitioners is 0.78 across hundreds of thousands of trainer professionals around the world.

Job-embedded coaching is the second strategy for moving person-centered and trauma-informed care from theory to practice. Coaching can be provided by clinical supervisors as part of their teaching and staff professional development role, or by having a separate coaching role embedded within programs or departments. Providing coaching and support on attaining fluency on the CANS items and action levels by reflecting on the client's story and updating the ratings as new information arises is a way to make the CANS a routine part of supervision or coaching sessions. Regular updates ensure that the young person's story is current. The CANS can be used in combination with established clinical interviewing strategies, such as motivational interviewing, and clinical skills, such as engagement and rapport building, having difficult and/or sensitive conversations with young people or families, identifying traumatic stress, and facilitating collaborative planning processes.

It has been our experience that by using a consensus approach to the assessment process, the CANS facilitates a healthy engagement (assessment *with* people rather than *of* people). Systems using the CANS have also reported that it can be a reliable means of keeping the focus of supervision and coaching on the broader understanding of the child and family's story. With feedback on items included in the plan interventions that may not be working optimally, changes to the plan due to detection of new traumas or traumatic stress behaviors, or progress that should be celebrated can be easily identified. Supervisors or job-embedded coaches can also help support staff on how to talk to young people and families about their conditions, their care, and their progress toward their goals.

In a teaming process such as a Child and Family Team or Treatment Team, the CANS can serve as a consensus-building tool that helps the team better understand the young person and family's needs and strengths as well as focus and coordinate their support. The team member's differing perspectives are welcome and clarification and consensus-building on the areas that require support and urgency of action can be facilitated through the use of the action levels of specific CANS items to facilitate a shared understanding and prioritization of the work ahead. Even children can participate in this process when the action levels are described and/or depicted in age-appropriate ways.

The CANS organizes and prioritizes information gleaned from the assessment process. The ratings on the items indicate where the action is needed: interventions, resources, and services to address needs; and building or development for strengths. By explicitly integrating the actionable items from the CANS into a collaborative planning process with individuals and families and tracking the plan over time through feedback reports from the updated CANS items, managing transformational change is now possible with young people and families as full participants in their care.

#### 3.1.2. Mass customization

**Figure 2:**
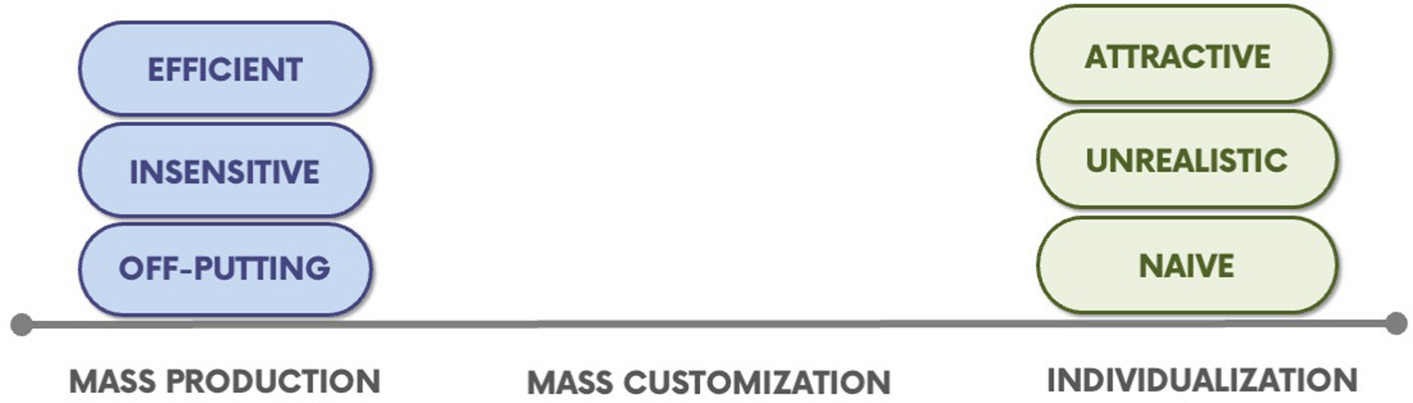
The continuum of helping: mass production, mass customization, and individualization (Praed Foundation, 2023).

A key guiding principle of TCOM is the idea that approaches to helping should be adjusted to reflect important differences across people. Arising from the work of Pine and Gilmore (2011), this principle is called “mass customization.” “Build a bear” is the favored example of mass customization by these authors (Pine and Gilmore, 2011). These economists make an argument that transformational offerings can be more effective if mass customization is used as an entry into treatment. As seen in Figure 2, mass customization falls on a continuum between Mass Production (i.e., treating everyone the same) and Individualization (i.e., treating everyone as completely different).

While we are unaware of substantial research on the differential effectiveness of Mass Production (standardized protocols of asking everyone the same question in the same order always) vs. more customized approaches to understanding people's stories, anyone who works in these systems knows that people prefer starting with their concerns rather than being asked a large number of potentially superfluous questions (Lyons, 2022). The research on engagement strategies is supportive of this proposition (e.g., Becker et al., 2017; Waid and Kelly, 2020).

Customization is one of the greatest challenges of system management in moving away from mass production models of service delivery and creating programs that can adjust interventions to fit the specific needs of specific people at specific times in their life journeys. Efficiency arises from creating processes of care and sticking to those processes—this mass production approach is a vestige of the industrial revolution (Note: child welfare is another vestige of industrialization as people moved from farms to the cities, and suddenly large families were less of a benefit and more of a burden). The application of mass production to the helping sector involves standard “intake” procedures, structured assessment processes, and formalized intervention strategies that everyone receives as a standard of a program. Economists from Smith (1776) to the present will tell you that this is the most efficient way to deliver care, at least if that care is about service delivery rather than personal change.

We believe that there are two fatal problems with mass production as it applies to trauma-informed systems of care. First, it can be experienced as dehumanizing. When helpers apply a standard approach to interacting with people as they enter care, it will invariably be experienced at least by some that the helper cares far more about their paperwork and bureaucracy than they do about the circumstances of the help-seeking person. This is one reason why Pine and Gilmore (2011) recommend mass customization to provide a more powerful personal experience. Second, mass production approaches will not work because there is usually substantial variation across people in any given program. For example, Ebesutani et al. (2017) have demonstrated that any three evidence-based practices in a community clinic will likely be appropriate for less than a third of the presenting challenges. Third, it is not trauma-informed. Treating everyone exactly the same assumes that they are the same which is simply not true.

Some people have proposed individualization as the solution to the problems of mass production. Calls for individualized care come from Wraparound and Recovery programs. Even schools refer to Individual Education Plans. However, individualization has an entirely new set of fatal challenges. The most important problem is that it is impossible and naïve to be “individualized.” If everyone were entirely different, then there would be nothing we could do to help. We would not learn anything from one person to the next. Education and training would be irrelevant because every situation is different and requires different actions. That is simply not true. We actually decide how to help based on identifying core commonalities. We use the items on the CANS to identify and assess these core commonalities that influence decisions about help. We understand and appreciate differences, but we help based on how people are the same. This is called mass customization.

### 3.2. Process component of a TCOM trauma-informed system

From a TCOM perspective, a trauma-informed system should have a set of processes in place to support the ongoing implementation of trauma-informed care within a trauma-informed system.

1. Detection of trauma experiences early in care.
2. Monitoring of traumatic stress symptoms regularly
3. Recognition that there is no need to force people to continually and repeatedly answer the same questions; however, people's stories unfold over time based on evolving trust and recognition.
4. An integrated approach to training, teaching, supervising, and monitoring the workforce to create a perpetually learning and sometimes re-learning environment.
5. Development of a differential response whereby the focus of standard, evidence-based interventions is tailored to the specific circumstances of the individual.
6. Use of aggregate data to inform system performance and highlight opportunities.

## 4. Practice-based research to support trauma-informed sectors

### 4.1. Trauma-informed child welfare

It has become clear that identifying and addressing the impact of trauma is a fundamental responsibility of an effective child welfare system (Akin et al., 2017). All children in out-of-home care have at minimum experienced the disruption of their caregiving environment (U.S. Department of Health Human Services, 2022). Many have experienced neglect (U.S. Department of Health Human Services, 2022) and others have had notable experiences with abuse (Euser et al., 2013). Multiple and complex trauma is commonplace (Greeson et al., 2011). Research on Adverse Childhood Experiences (ACEs) suggests that there may be a “dose–response” effect of trauma experiences where increasing exposure is predictive of greater life impact (Briere et al., 2008; Oral et al., 2016).

While the frequency of traumatic experiences in child welfare populations is well-studied, less is known about the expression of traumatic stress symptoms between these children and youth. While the impact of trauma experiences on functioning and other child welfare outcomes has received substantial attention, the clinical pathways to these higher-level outcomes are less known. Still less is known about how the child welfare system detects traumatic stress symptoms so that they can initiate trauma-informed treatments. Some systems are even trying to build referrals directly based on trauma exposure without understanding the child's lived experience (Verbist et al., 2020). While many children and youth who experience traumatic events develop trauma stress systems, it appears that this relationship is not uniform (Nilsson et al., 2022). Understanding the potentially complex differences between children and youth who *do* and *do not* express traumatic stress symptoms in the face of traumatic experiences is an important direction of research to inform practice and policy.

In a person-centered, mass customized (TCOM) approach to trauma-informed care in child welfare, intervention is based on evidence of an effect of those experiences—the symptoms of traumatic stress. Providing trauma treatment for children without symptoms of traumatic stress might even have the potential to re-traumatizing the child and creating traumatic stress when there was otherwise none.

To clarify how traumatic experiences and traumatic stress fit together, we have undertaken a program of research to explicate the potentially complex relationship among these important constructs for a trauma-informed child welfare system. Using a cross-section of initial CANS assessments on a series of children recently taken into custody in a mid-sized state child welfare system, we looked at the relationship between the presence of traumatic experiences and the detection of symptoms of traumatic stress (Tumlin et al., in press). This sample was 53% male, 57% White, and 32% African American. In this state, the average user reliability at training on the CANS is 0.78. Average recertification reliability, taken annually, is generally higher.

**Figure 3:**
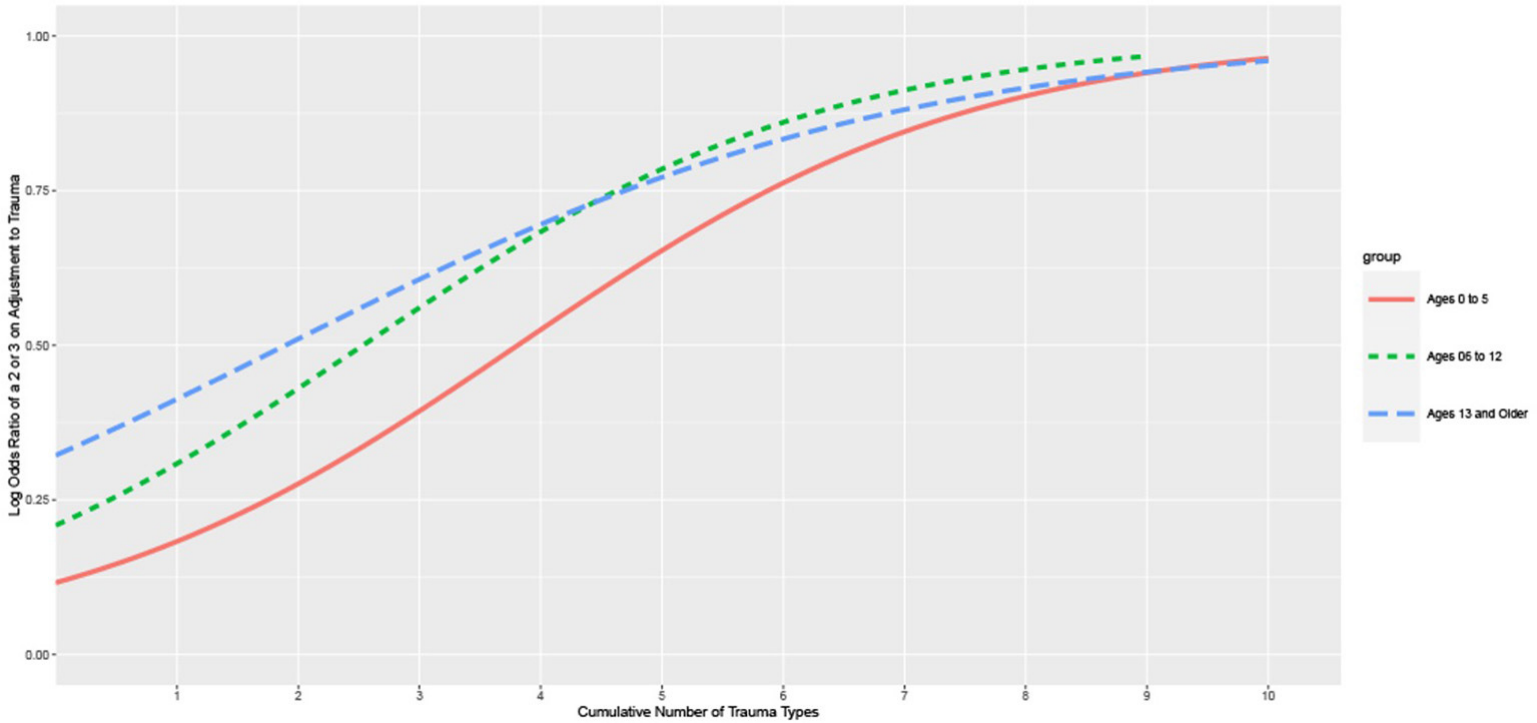
Logistic regression of the number of trauma types predicting actional adjustment to trauma ratings: the Child and Adolescent Needs and Strengths (CANS) comprehensive assessment data from a Child Welfare System in a Midwestern State, 2011–2021.

Figure 3 presents the results of a logistic regression of the relationship between the total number of ACEs and the likelihood of the children presenting with “actionable” Adjustment to Trauma (ratings of 2 or 3), which is the CANS item that describes the presence of traumatic stress symptoms. Children “above the curve” are not seen as having traumatic stress reactions and those “below the curve” are seen as expressing symptoms of traumatic stress. This figure highlights the relationship between the number of traumatic experiences and the likelihood that children aged 5 years or younger would be identified as experiencing one of four traumatic stress symptoms. Children and youth experiencing six or more traumatic experiences very often are expressing traumatic stress symptoms.

For all age groups, this graph demonstrates that the more cumulative trauma experienced, the greater the likelihood that they are seen to be also experiencing symptoms of traumatic stress. Notice that the detection rate (i.e., the likelihood of a child/youth being described as having symptoms of traumatic stress) was steeper for older children and youth. Young children had the lowest rate of detection of traumatic stress with the rising number of traumatic experiences. This graph also demonstrates that the majority of children with three or fewer traumatic experiences are not seen as having symptoms of traumatic stress. It is not until the child has experienced seven or eight different traumatic experiences (i.e., ACES) that they are routinely reported as having symptoms of traumatic stress. There are three possibilities for this phenomenon:

° Traumatic stress is missed.
° Traumatic stress will unfold over time but is not currently observable.
° The child is resilient and will not experience traumatic stress despite their traumatic experiences.

These three possible types of children would require dramatically different policy and practice solutions should they explain this relationship. Failing to detect traumatic stress suggests training and supervision challenges. Unfolding traumatic stress calls for a prevention/early intervention solution. Resilient children should be celebrated and the things that help them be resilient should be supported. Providing trauma treatment for these children seems like a very bad idea to these authors.

To better understand children with notable trauma experiences who did and did not express symptoms of traumatic stress, we did a follow-up analysis with the youngest children (i.e., 5 years old and younger). Furthermore, to create a cohort of young children who would reasonably be expected to have these systems, we identified only those children in the population who had complex interpersonal trauma (CIT). Kisiel et al. (2009) among others have defined CIT in the literature as children having at least two of the following interpersonal trauma experiences: neglect, sexual abuse, physical abuse, emotional abuse, and witness to family Violence. We further reduced the sample to only those children who were not observed to have symptoms of traumatic stress. A latent class analysis was used to group these children, and using the convergence of the Akaike Information Criterion (AIC) and Bayesian Information Criterion (BIC) curves, a three-class solution was observed. Analysis of these classes using the items of the CANS revealed that the largest class, comprising about 50% of the young children in the sample had good strengths and few needs. These children could reasonably be called resilient in the face of their traumatic experiences.

It was noteworthy that the other two classes of children without traumatic stress had distinct clinical presentations. One group consisted of children with medical trauma and medical and developmental needs but few behavioral or emotional needs. We believe that these children might represent a group whose traumatic stress symptoms might unfold over time. The third group was more likely boys and had a great deal of externalizing behaviors. We think these children are the ones whose traumatic stress symptoms were simply missed.

### 4.2. Trauma-informed behavioral health

There is every reason to believe that an approach to creating a trauma-informed behavioral health system from a TCOM perspective might be different than that required in a child welfare system. The most salient difference is that children enter the child welfare system, usually, although not always because of the behavior of others—primarily their parents and/or caregivers. In the behavioral health system, generally, children are referred for help *because* of concerns about their behavior. There is far more reason to suspect that unidentified traumatic stress in the face of notable traumatic experiences is a function of a failure to detect or an unfolding process of the child's resiliency in the face of adverse childhood experiences.

**Figure 4:**
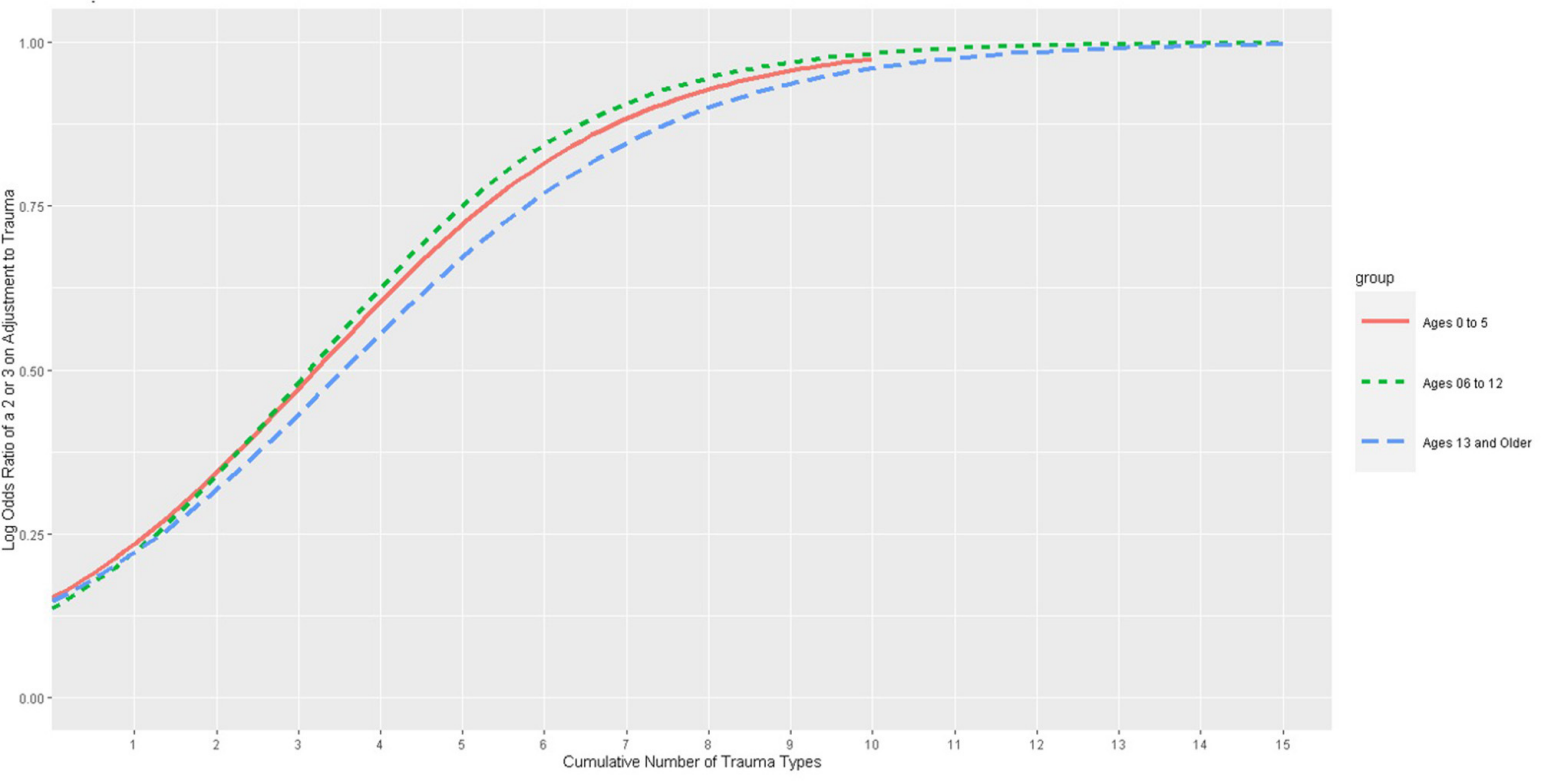
Logistic regression of the number of trauma types predicting actional adjustment to trauma ratings: the Child and Adolescent Needs and Strengths (CANS) comprehensive assessment data from a Behavioral Health System in a Pacific Northwestern State, 2021–2022.

Figure 4 presents the logistic regression between the number of traumatic experiences and the likelihood of identified traumatic stress. The sample for this analysis was 34,411 children presenting for public behavioral healthcare: 51% male, 81% White, and 15% Native American. The average reliability of all actively certified CANS users in this state is 0.80. Notice in this graph, the opposite relationship between age and detection rate is observed. Older youth lag behind younger ones. In other words, young children are more likely than older children to be seen as having traumatic stress with an increasing number of traumatic experiences. It is not until the overall number of traumatic experiences is high that older youth experience the same detection rate.

For these analyses, since older youth lagged behind younger, we completed an LCA on the older age group that had experienced CIT. Thus, the relationship between age and traumatic stress detection is reversed between behavioral health and child welfare. For child welfare, young children are less likely to have detected traumatic stress. In behavioral health, it is adolescents who lag in the detection of traumatic stress.

Focusing on the adolescents, a latent class analysis revealed a two-class solution with one class more frequently represented by girls and internalizing symptoms and the other class characterized more frequently by boys engaged in externalizing behaviors. Although further research is needed, we believe that both of these classes represent different forms of “misses.” The absence of a “resilient” class in this population likely reflects the fact that these were assessments exclusively of children and youth seeking behavioral health treatment.

## 5. Summary and next steps

The purpose of this study was to describe an evolving TCOM strategy for the creation of an assessment-based infrastructure to support the evolution of a trauma-informed system of care. In our work to date, we have made some strides toward this aspiration. Over 40 statewide implementations spanning 25 years, with the CANS touching millions of children and youth, we have demonstrated that it is possible to create and implement the consistent use of a person-centered assessment process that provides reliable information about the needs and strengths of children, youth, and families in a fashion that informs the understanding of individual trauma histories and monitors traumatic stress. In this study, we report data on about 50,000 children in two states with an average reliability of at least 0.78. Worldwide, the CANS is used by more than 10 million children and families each year (Lyons, 2022). We have also demonstrated the potential value of using system-level data to support our understanding of the system's performance in the detection of traumatic stress symptoms in two different settings—child welfare and behavioral health. Once in place, this infrastructure can be used to support more effective trauma-informed practices at both the individual and system levels.

Of course, there is much more work to be done. It is important to expand this work to more culturally diverse settings and populations to understand whether resilience or the lens for detecting traumatic stress symptoms is influenced by culture. Also, it will be necessary to confirm that the “unfolding” group of children and youth do, in fact, manifest traumatic stress later. And if so, identifying effective interventions that help these children and youth reach resiliency without ever having to endure traumatic stress symptoms is important. Furthermore, while evidence-based trauma interventions are already available for those children and youth with identified traumatic stress, more work is required to ensure access and consistent effectiveness. Additional training and supervisory support are necessary to reduce the number of children and youth whose traumatic stress is undetected because of a misinterpretation of externalizing behavior. Business models that sustain skilled workforces to meet the needs of highly traumatized children are also critical. Furthermore, we need to build and implement system-wide strength-building approaches and test whether strength-building/resilience proves to be preventive of traumatic stress in the face of traumatic experiences over time.

Many systems are moving from a child focus to conceptualizing the work from a family perspective. As this evolution unfolds, it will be important to conceptualize a trauma-informed system that is family-based (c.f. Lee et al., 2020). For this reason, we have developed a trauma-informed version of the Family Advocacy and Support Tool (FAST, Lyons, 2022). The FAST Conceptualizes the measurement of the story of the family rather than a single individual. This tool provides an assessment infrastructure to support systemic shifts to family-based approaches.

Real change is glacial (Lyons, 2004). The work we presented in this chapter has occurred over the past decade in two states. Similar work is being done all over the country. Creating infrastructure for a person-centered system of care is neither easy nor quick. It requires a steady dedication to ensuring that the stories of the people we serve are discovered respectfully and flexibly. It requires that these stories are coded and communicated with integrity and reliability, and it requires that the information from these stories be used creatively to inform decision-making at the individual, program, and system levels. We owe the people who seek our help nothing less.

## Data availability statement

The original contributions presented in the study are included in the article/supplementary material, further inquiries can be directed to the corresponding author. Requests to access these datasets should be directed at: iphcenter@uky.edu.

## Author contributions

JL and AF contributed to conception and design of the paper. JL wrote the first draft of the manuscript. AF wrote sections of the manuscript and created some of the figures. Both authors contributed to manuscript revision, read, and approved the submitted version.
